# Multifunctional nanoparticles for real-time evaluation of toxicity during fetal development

**DOI:** 10.1371/journal.pone.0192474

**Published:** 2018-02-08

**Authors:** Sean Sweeney, Andrea Adamcakova-Dodd, Peter S. Thorne, Jose G. Assouline

**Affiliations:** 1 Department of Biomedical Engineering, University of Iowa, Iowa City, IA, United States of America; 2 NanoMedTrix, LLC, Coralville, IA, United States of America; 3 Environmental Health Sciences Research Center, University of Iowa, Iowa City, IA, United States of America; 4 Department of Occupational and Environmental Health, University of Iowa, Iowa City, IA, United States of America; Otto von Guericke Universitat Magdeburg, GERMANY

## Abstract

Increasing production of nanomaterials in industrial quantities has led to public health concerns regarding exposure, particularly among pregnant women and developing fetuses. Information regarding the barrier capacity of the placenta for various nanomaterials is limited due to challenges working with *ex vivo* human placentas or *in vivo* animal models. To facilitate real-time *in vivo* imaging of placental transport, we have developed a novel, multifunctional nanoparticle, based on a core of mesoporous silica nanoparticles (MSN), and functionalized for magnetic resonance imaging (MRI), ultrasound, and fluorescent microscopy. Our MSN particles were tested as a tracking method for harmful and toxic nanomaterials. In gravid mice, intravenous injections of MSN were administered in the maternal circulation in early gestation (day 9) and late gestation (day 14). MRI and ultrasound were used to track the MSN following the injections. Changes in contrast relative to control mice indicated that MSN were observed in the embryos of mice following early gestation injections, while MSN were excluded from the embryo by the placenta following late gestation injections. The timing of transplacental barrier porosity is consistent with the notion that in mice there is a progressive increasing segregation by the placenta in later gestation. In addition, built-in physico-chemical properties of our MSN may present options for the therapeutic treatment of embryonic exposure. For example, if preventive measures such as detoxification of harmful compounds are implemented, the particle size and exposure timing can be tailored to selectively distribute to the maternal side of the trophoblast or delivered to the fetus.

## Introduction

The widespread and ever-increasing manufacturing of industrial, pharmaceutical and food products incorporating engineered nanoparticles (NP) is of concern to public health. As mesoporous silica nanoparticles (MSN) and other metal oxides take an increasingly important role as an integral part of building infrastructure [1] and the medical drug process [2,3], the risks of exposure increase in kind. Pregnant women and developing embryos/fetuses comprise a particularly vulnerable population, as NPs that infiltrate the bloodstream encounter barriers including the placenta [4–7]. At these locations, the NP have the potential to cause inflammation or other tissue damage [8,9], yet in the case of the placenta information regarding the barrier capacity is limited, due mostly to technical obstacles and ethical issues that present difficulties in the study of the role of the placenta in both fetal and child development. As a result, little is known about the translocation parameters governing the exchanges between mother and fetus, or about the impact of environmental pollutants and particulate contaminants.

The placenta performs a wide range of physiological functions; impairment of its proper functioning will have potentially irreversible prenatal and postnatal effects and possibly life-long impacts on human adult health. In recent years, the number of studies investigating transport of nanomaterials through the placenta [10,11], and their effects on the fetus [5,12,13] is significantly increasing. An *ex vivo* study of the barrier capacity of human placenta found that polystyrene particles (50–500 nm) were taken up by the placenta and were able to cross the placental barrier [11]. Also, an *in vivo* study in mice showed that quantum dots crossed the placental barrier [5]. Some studies have investigated the possibility of translocation of NPs to fetus and the effects of NPs on the developing fetus after i.v. [14,15] or subcutaneous [16,17] administration to pregnant dams. Silica and titanium dioxide NPs (with primary diameters of 70 and 35 nm, respectively) were found in the placenta, fetal liver and brain after i.v. administration [15]. Other studies focused their research on *in utero* effects of NPs after inhalation exposure [12,13,18]. A vast literature suggests the permeability of the placenta has time dependence and size dependence [19,20].

In animal models, the yolk sac plays a more important role in early embryogenesis than in humans. In mouse models, the developing placenta becomes less permeable to foreign objects of most sizes after gestational day 11, while in rabbits this occurs on gestational day 13, analogous to Carnegie stage 13, approximately 30 days in human embryonic development [4]. After this time, the placenta becomes less permeable to foreign objects of most sizes. In late-term human gestation, for which small animals are a poor model, the placenta becomes more permeable [21]. Studies suggest that clathrin-mediated endocytosis is the preferred method of internalizing immunoglobulins, insulin and transcobalamin-vitamin B12 complexes into the fetal circulation [19,20]. Similar endocytotic studies have also reported nanoparticle internalization in several models [22,23], as well as across the transplacental barrier [15,24].

Taken together, the study of the placental function in optimizing fetal development is hindered significantly by technological and ethical challenges. To address these challenges, our group has developed a multifunctional nanodevice with a core of mesoporous silica (MSN), engineered to provide real-time reporting of changes during pregnancy. The particle diameter ranges from 50 nm to 187 nm, depending on its composition, and a hexagonal array of pores 2.4 nm in diameter. In addition the MSN has been functionalized for use in ultrasonography as well as magnetic resonance imaging (MRI). While sonography is the most common modality for imaging the placenta/fetus, MRI is increasingly utilized in both the lab animal and in human subjects [25,26]. Our MSN take advantage of the features of dynamic contrast-enhanced MRI (DCE-MRI).

In DCE-MRI, the use of gadolinium- or iron oxide-based contrast agents enhances visualization of the vasculature [26], which helps reveal abnormal blood flow conditions within the placenta layers [27]. In one study, gadolinium chelates were conjugated to the large protein albumin, which does not cross the placental barrier, as a means to measure blood turnover rates in the maternal compartment of the placenta in normal [28] and PKBα/AKT1 (a mediator of angiogenesis) knockout mice [29].

Our overall goal is to use our MSN to advance the knowledge of placenta/fetal interdependence in health and in environmental exposure studies. In this report, we present data demonstrating the temporal detection of MSN transfer across the placental barrier. Using a mouse model for pregnancy, particles injected into the maternal circulation were detected in the placenta and fetal circulation in a time-dependent manner. We predict applicability in clinically relevant imaging modalities (MRI, ultrasound) as well as in study in nano-size toxicant in animal models. Given that our particle compounds are not toxic based on systematic studies [30], we envision the covalent linking of difficult to track environmental pollutants of interest, and use of this technology as vehicles for real-time, non-invasive studies.

## Methods

### Particle synthesis and characterization

First, a gadolinium oxide-MSN core was manufactured as reported previously [30–32], yielding 1.79 grams. Next, FITC (5.0 mg, 0.0128 mmol) was reacted with aminopropyltrimethoxysilane (2.2345 μL, 0.0128 mmol) in DMSO (1 mL) for 2 hours, and FITC-Gd_2_O_3_-MSN was prepared by grafting 0.05 mL of the resulting product on the previously synthesized Gd_2_O_3_-MSN (100 mg) in toluene (25 mL) under reflux for 24 hours. The resulting solution was filtered and the obtained yellow solid was washed with methanol. Finally, the particles were further functionalized with trifluoropropyl (TFP) moieties by grafting trimethoxy (3,3,3-trifluoropropyl) silane (91.6 μL, 0.48 mmol) on FITC-Gd_2_O_3_-MSN (100 mg) in toluene (25 mL) under reflux for 24 hours. The resulting solution was filtered and the obtained yellow solid (TFP-FITC-Gd_2_O_3_-MSN) was washed with methanol, dried under vacuum, and stored at room temperature. The materials were characterized by X-ray diffraction, using a Rigaku Ultima IV diffractometer, nitrogen sorption analysis in a Micromeritics ASAP 2020 surface area and porosity analyzer using the Brunauer-Emmett-Teller equation to calculate surface area and pore volume and the Barrett-Joyner-Halenda equation to calculate the pore size distribution. Dynamic light scattering (DLS) was used to obtain particle size distribution and zeta potential data, using the Malvern Zetasizer Nano ZS instrument. Samples were dispersed in ethanol and the instrument performed 3 sets of 13 runs on each sample. The 3 sets were averaged by their number-based distribution. The materials were also visualized by transmission electron microscopy (TEM) by supporting samples on copper grids in a Tecnai G2 F20 microscope operating at 200 kV.

### Animal models

All procedures were performed according to NIH guidelines and previously approved by the Institutional Animal Care and Use Committee (IACUC) of the University of Iowa. C57Bl/6 mice (Jackson Laboratories) were acclimatized over several days before beginning the study in our vivarium in polypropylene, fiber-covered cages in HEPA-filtered Thoren caging units (Hazelton, PA). Food (sterile Teklad 5% stock diet, Harlan, Madison, WI) and water (via an automated watering system) were provided ad libitum. Light–dark cycle (12 hr) was maintained in the animal room. Female mice were housed over 1–3 days with males, and removed after pregnancy was visually confirmed by the presence of a vaginal plug. Gravid mice were injected intravenously with TFP-FITC-Gd_2_O_3_-MSN (1 mg/0.1 mL normal saline) or vehicle alone (0.1 mL normal saline) using a tail vein approach. Dams were injected during early-mid gestation (GD 7–9) or late gestation (GD 14–15), with MRI and ultrasound image acquisitions occurring at times before and after particle injection. Prior to delivery (GD 16–17), dams were euthanized by CO_2_ asphyxiation followed by cervical dislocation, in accordance with institutional and NIH guidelines, and organs were weighed, measured, and processed for histopathology. Maternal sera were assed for cytokines and reactive oxygen species/reactive nitrogen species.

### MRI and computational image processing

Mice were injected with TFP-FITC-Gd_2_O_3_-MSN (1 mg/0.1 mL normal saline) or with normal saline alone as described above. After injections, the mice were anesthetized with inhaled isoflurane and scanned using the Varian® 4.7T small animal MRI scanner with a 25 mm RF coil. Axial T_2_-weighted fast spin echo multislice MRI scans were acquired (repetition time 3200 ms, echo time 15 ms, image size 256 x 256 x 29 pixels, 0.156 x 0.156 x 0.45 mm pixel size). Raw MR images were processed through a method utilizing partial volume reduction through reverse diffusion [33,34] and using in plane and out of plane anisotropic diffusion modules in the 3D Slicer, as well as preprocessing using an in-house MATLAB algorithm. Intensities were normalized between segmentations by dividing each segmented image by the mean intensity of the hyper-intense fat region surrounding the embryos in preprocessed image and by multiplying each image by a factor of 1000. Embryos and placentas were manually segmented using built-in tools in MIPAV and 3D Slicer.

### Ultrasound and signal processing

Mice were sedated with 0.1 mL midazolam delivered subcutaneously and injected with TFP-FITC-Gd_2_O_3_-MSN. Within 2 minutes before and after injection, mice were scanned using the VEVO® 2100 small animal ultrasound system and a 30 MHz probe with 12 mm depth of penetration. Images were acquired using normalized parameters for gain, and saved as individual images or 3 second, 30 frame cine loops. Frames were manually segmented and analyzed using MIPAV software.

### Statistical analyses

Experimental group sizes were determined from calculations of statistical power based on relevant past experiments. To achieve a significant statistical power level of 90%, a minimum of 3 mice per study group were used. Up to 6 mice per study group were used to account for non-gravid dams. Comparisons of measurements between groups of mice were made using Welch’s method for the Student’s unpaired T-test with populations of unequal variances, with an alpha level of 0.05 considered significant. The average litter size was 7.5 pups, and each placenta/embryo was treated as an independent datum; these were then pooled together with all dams of a given experimental condition. Comparisons of volumes within the same mouse were made using a two one-sided t-test (TOST) [35] with a significance level of α = 0.05 and a test margin of 0.05, a unitless value of normalized MRI intensity representing the average smallest difference between two intensities that our image processing technicians could visually discern.

## Results

### Particle characterization

**Fig 1:**
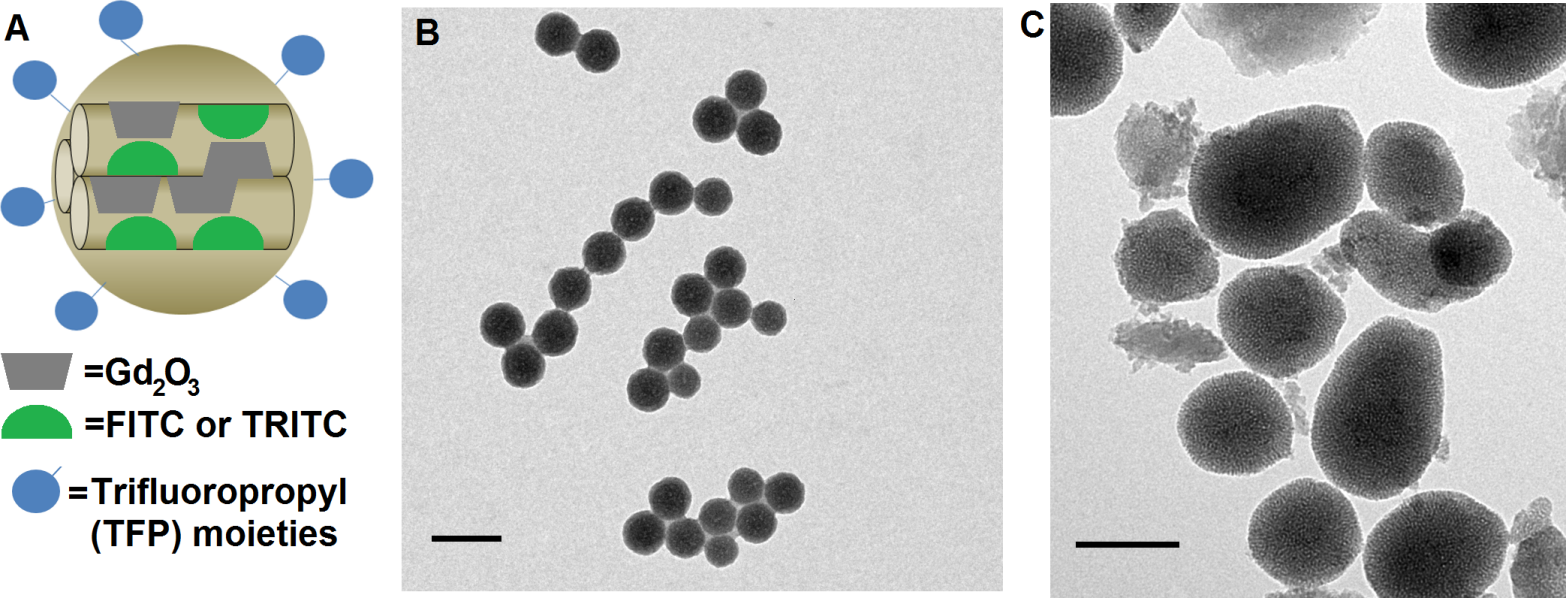
Characterization of mesoporous silica nanomaterials (MSN). (A) Schematic describing the particle, with Gd_2_O_3_ and FITC or TRITC incorporated into the silica matrix and functionalized with trifluoropropyl (TFP) moieties. (B) Transmission electron micrograph of synthesized MSN, and (C) TFP-FITC-Gd_2_O_3_-MSN. Scale bars equal 100 nm.

To ensure a homogeneous particle size and morphology, the particles were characterized after each step in the synthesis and were reported previously [30–32]. Briefly, the Gd_2_O_3_ colloid prepared in diethylene glycol was measured by DLS, having a number PSD-based radius of 7.4 nm and a polydispersity index of 0.209. Transmission electron micrographs show areas of increased density within the silica matrix of the Gd_2_O_3_-MSN (Fig 1C), not present in the plain MSN (Fig 1B), indicative of incorporation of Gd_2_O_3_. Preliminary MRI of standardized phantoms containing relevant concentrations of TFP-FITC-Gd_2_O_3_-MSN showed concentration-dependent T1- and T2-relaxivity, further confirming the presence of Gd_2_O_3_ in the silica. When compared with MSN not bearing trifluoropropyl groups, the TFP-FITC-Gd_2_O_3_-MSN agars also generated a clear ultrasound contrast. Additionally, we observed contrast changes *in vivo* when previously evaluating the biocompatibility of the particles [30]. Powder XRD analysis confirmed ordered pores in the diffraction pattern of the Gd_2_O_3_-MSN as evident by an intense d_100_ peak at 2.5 2θ, and well resolved d_110_ and d_200_ peaks at 3.83 and 4.40 2θ, respectively. This diffraction pattern is consistent with P6 mm hexagonal symmetry characteristic for MSN [36,37]. To verify the overall structure we performed TEM of the Gd_2_O_3_-MSN particles, confirming the expected hexagonal pattern as well as uniform size distributions and good dispersibility with little aggregation (Fig 1). After synthesis, the particles are washed copiously with methanol to remove organically soluble impurities: excess fluorophores, CTAB surfactant, and trifluoropropyl silanes. Insoluble materials may remain and include small, incompletely formed particles of amorphous silica, examples of which can be seen in Fig 1C. These materials make up a tiny fraction, both in size and number, of the total particle population and will be bioinert when applied *in vivo*.

The original synthesis of the Gd_2_O_3_-MSN core yielded over 1 g of material. From this, we allocated portions and grafted FITC onto one portion and TRITC onto another for use in multiple studies. For this study, we further aliquoted 20 mg of each for the addition of trifluoropropyl functionalization. Further measurement using nitrogen sorption analysis of the FITC-Gd_2_O_3_-MSN exhibited a Type-IV isotherm, typical of mesoporous materials, with a BET surface area of 710 m^2^g^-1^. The average pore diameter for TRITC-Gd_2_O_3_-MSN by BJH calculation was 24 Å [30]. Characterization of the size and dispersibility of the Gd_2_O_3_-MSN core and the complete TFP-FITC-Gd_2_O_3_-MSN was accomplished by dynamic light scattering (DLS). The Gd_2_O_3_-MSN core had a mean hydrodynamic diameter of 187 nm, while the complete TFP-FITC-Gd_2_O_3_-MSN had a mean hydrodynamic diameter of 227.8 nm. In deionized water, the FITC-Gd_2_O_3_-MSN had a zeta potential of -43.3 mV, becoming -7.12 mV after functionalization with trifluoropropyl groups [30].

### MRI quantitative evaluation of early gestational injections (GD 9)

**Fig 2:**
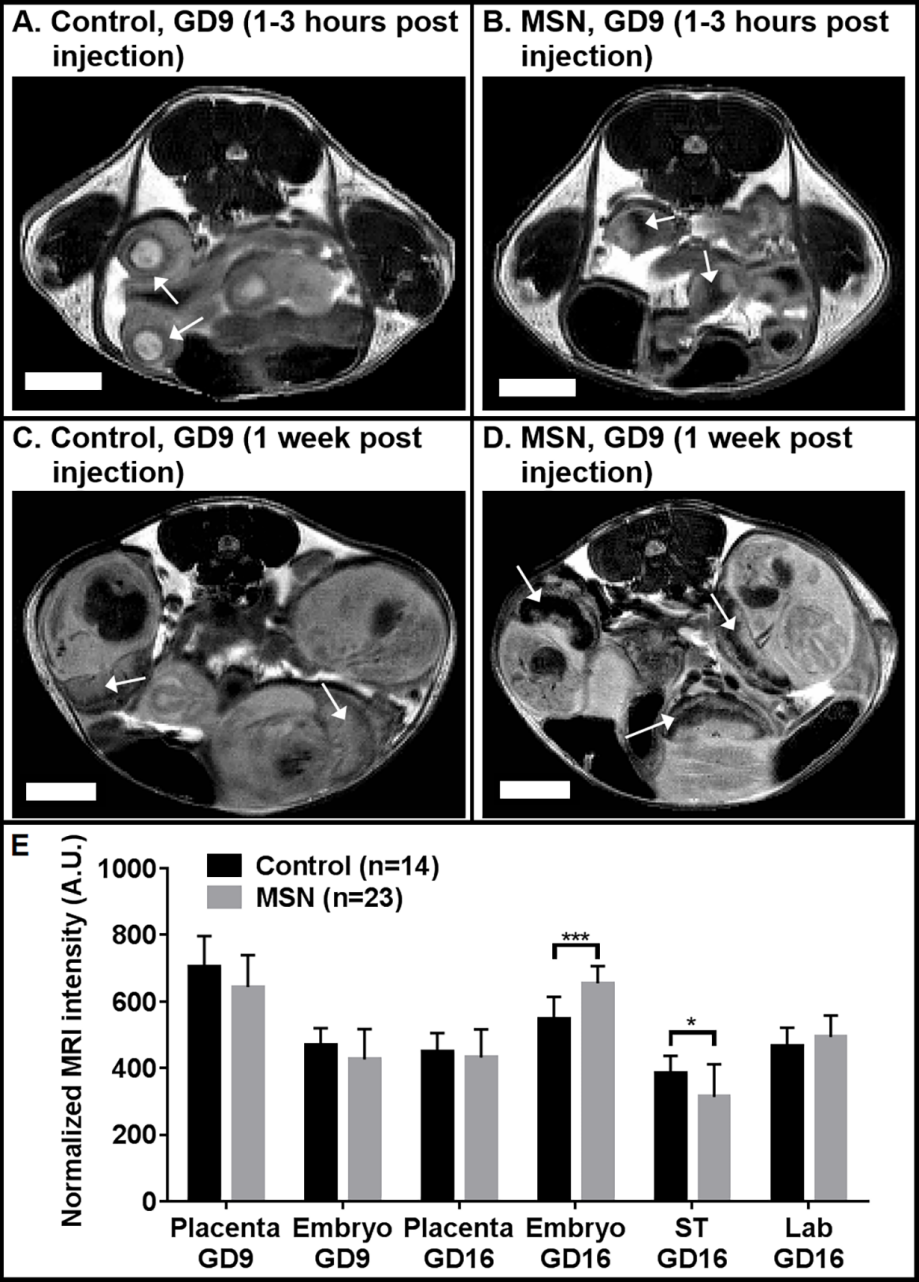
Representative slices of fat-normalized, T_2_-weighted MRI scans of gravid mice exposed to MSN injected early-mid gestation (GD 9). Within 3 hours of tail vein injection of normal saline (A) or MSN (B), placentas/embryos (arrows) have a noticeable hypointensity for the MSN mouse relative to the control mouse, indicating accumulation of MSN in both embryo and placenta. Seven days after injection (C, D), the placentas, specifically the spongiotrophoblast (ST) layer relative to the labyrinthine layer (Lab), of MSN-exposed mice are noticeably darker than normal saline controls, indicating accumulation of MSN in the fetal portion of the placenta. In the plot (E), the mean normalized grayscale values for each volume are shown. Scale bars equal 5 mm. Error bars indicate standard deviations; *p<0.05; ***p<0.0001. For control mice, n = 14 embryos/placentas in 2 pregnant dams; for MSN-exposed mice, n = 23 embryos/placentas in 3 pregnant dams.

Within 4 hours of intravenous injections of TFP-FITC-Gd_2_O_3_-MSN or normal saline (sham) in the maternal circulation, T_2_-weighted MRI scans were acquired which were then reconstructed, background masked, and fat-normalized. Contrast agents based on Gd_2_O_3_ generate a hypointensity when used with T_2_-weighting [38,39]. The placentas and embryos were manually segmented, with the normalized grayscale value and volume measured for each embryo separately. For early stage (GD9) injections, when individual mice were compared, the average difference between both the placentas and the embryos was found to be statistically significant (p<0.0001 and p<0.0005, respectively), and the placenta/embryos appeared to be noticeably hypointense relative to controls (Fig 2A and 2B), indicating an accumulation of MSN in both the embryo and placenta. In follow-up scans of the same mice performed on GD16, hypointensity was observed in the placenta (specifically the spongiotrophoblast layer; p<0.05), but not the embryo, indicating accumulation of MSN in the fetal portion of the placenta (Fig 2C and 2D).

### MRI quantitative evaluation for late gestational injections (after day 14)

**Fig 3:**
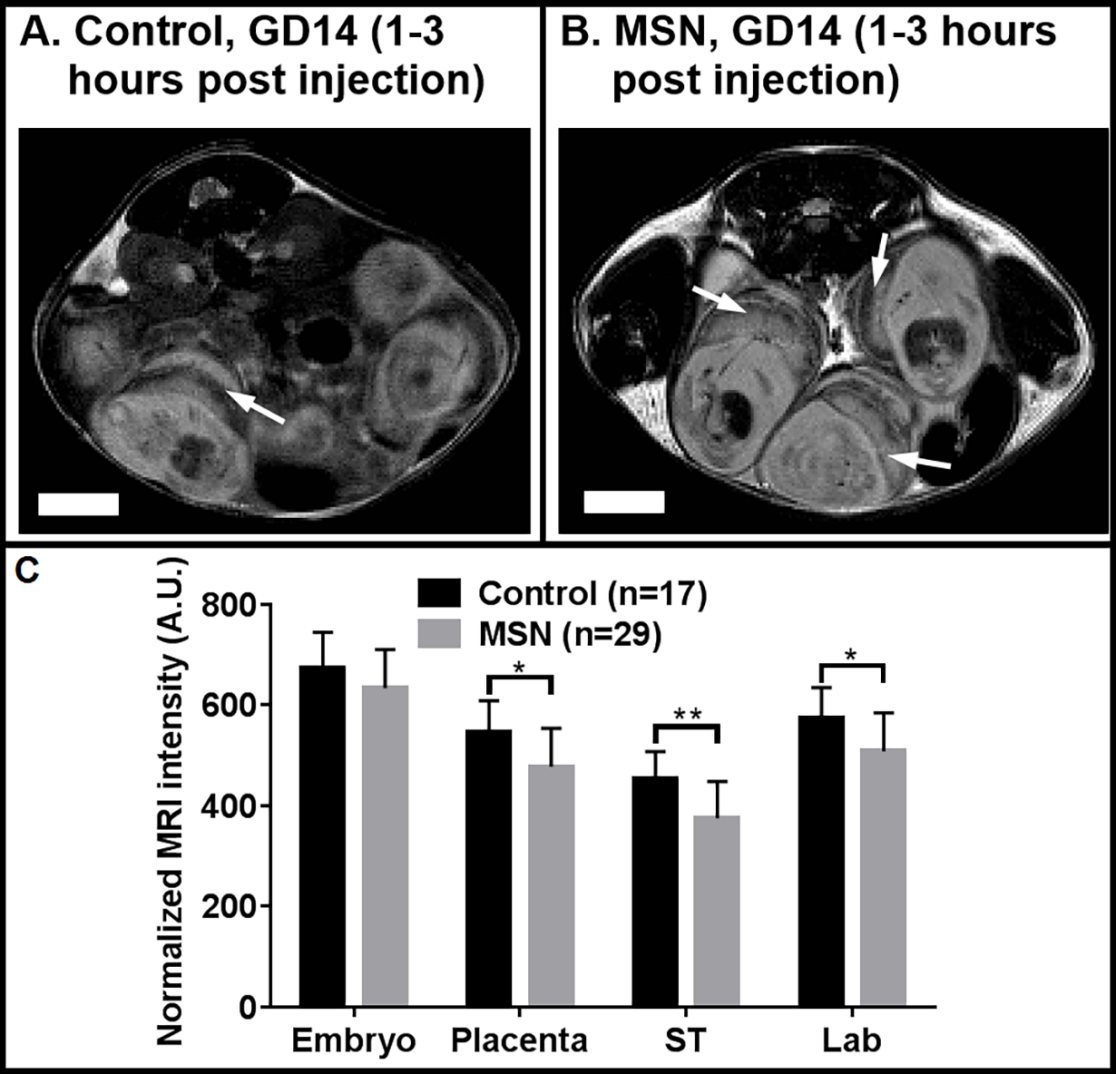
Representative slices of fat-normalized, T_2_-weighted MRI scans of gravid mice exposed to MSN injected during late gestation (GD 14). Relative to control mice (A), MSN-exposed mice (B) have significantly hypointense placentas (arrows) but not embryos. Hypointensity is observed in both spongiotrophoblast (ST) and labyrinthine (Lab) layers of the placenta. The plot (C) compared mean normalized intensities for each segment. Scale bars equal 5 mm. Error bars indicate standard deviation; *p<0.005; **p<0.0005. For control mice, n = 17 embryos/placentas in 2 pregnant dams; for MSN-exposed groups, n = 29 embryos/placentas in 4 pregnant dams.

Late stage (GD14) injections follow the anticipated trend that, due to the placenta becoming more exclusive, particles are not expected to infiltrate or transit through the placenta into the embryo (Fig 3). When embryos of all the mice are pooled (4 MSN and 2 controls), the intensity difference between the MSN and control placentas are significantly different (p<0.005), indicating placental accumulation of MSN, while the intensities in MSN and control embryos are equivalent.

### MSN localization by placental layer

**Fig 4:**
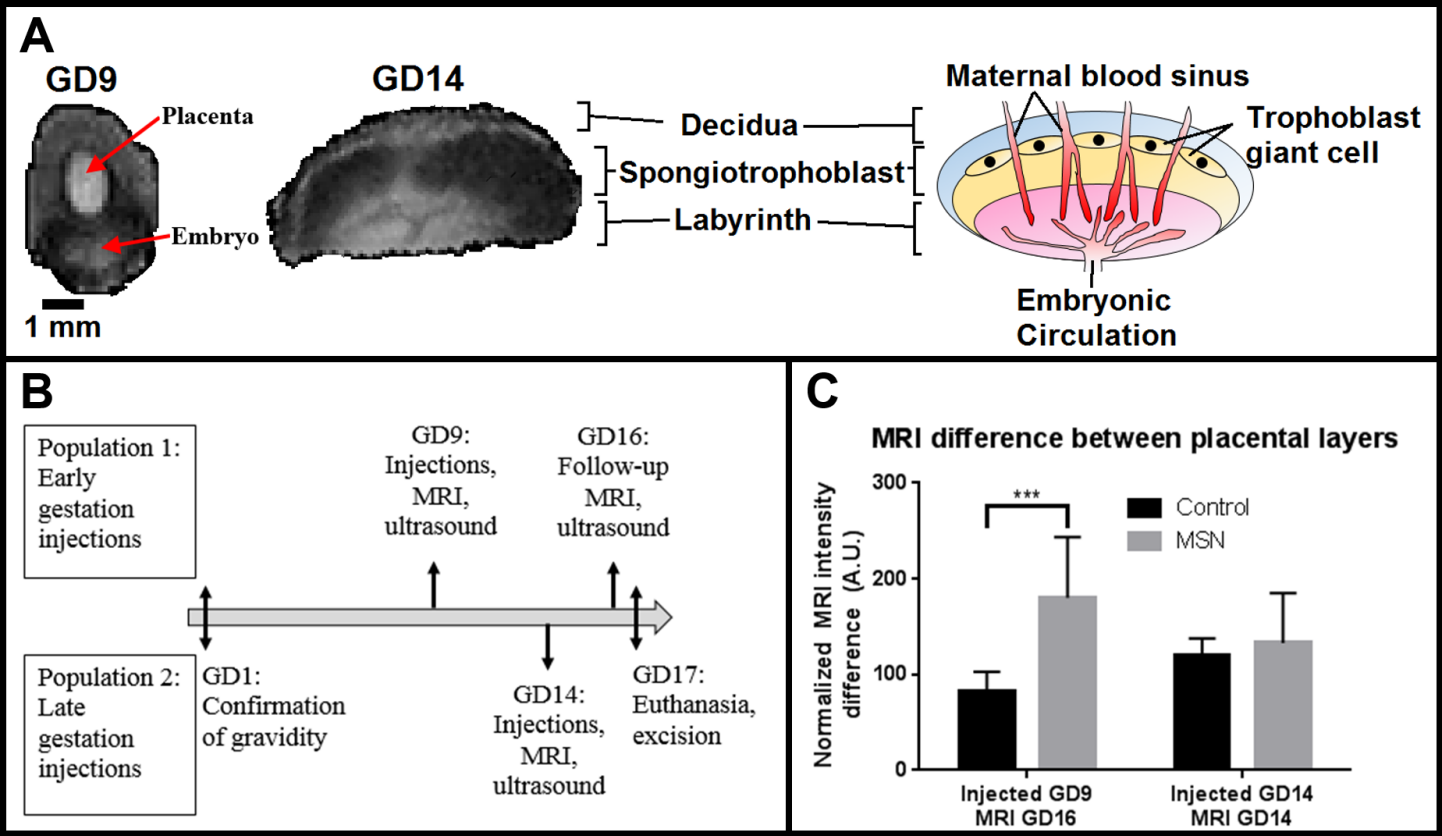
Engineered MSNs facilitate measurement of relative changes in individual layers of the placenta. (A) MRI placenta from GD 9 and GD14, showing the distinction between layers including the decidua (of uterine origin), spongiotrophoblast (ST, of fetal origin), and labyrinthine layer (Lab, location of blood exchange). (B) To evaluate the early gestation placentas with differentiable layers, the gravid mice were re-scanned on GD16 (↑-assessment of population 1, ↓-assessment of population 2, ↕-action on both populations). (C) On GD 16, the difference between the layers is significant for MSN-exposed mice relative to controls (p<0.0001), indicating a build-up of particles at the interface between the layers. On GD14, the difference between the layers is not significant for MSN-exposed mice relative to controls, suggesting the particles are not accumulating in any boundary of the placenta, rather they are in the maternal circulation. Error bars indicate standard deviation (n = 14 for GD9 control injections, 23 for GD9 MSN injections, 17 for GD14 control injections, and 29 for GD14 MSN injections).

A key feature of the placenta is the way in which the maternal and embryonic circulation interact. The placenta has 3 distinct histological layers: the decidua, comprising the uterine layer that gives rise to the maternal placenta, the spongiotrophoblast comprised mostly of embryonic circulation, and the labyrinth, comprised mostly of maternal circulation. In the GD14 and GD16 embryos, the spongiotrophoblast and labyrinthine layers are clearly differentiable in the MRI scans (Fig 4A), allowing us to segment these two layers and determine if the MSN reside primarily in the embryonic or maternal portion of the placenta. Under the early injection (GD9) paradigm, the GD16 follow-up scans were used to determine in which layer the particles reside after a week of circulation (Fig 2E). A significantly lower T_2_ intensity was observed in spongiotrophoblast (p<0.05), but not for the labyrinth layer in MSN versus control mice. Additionally, the intensity difference between the layers was significantly higher in MSN-exposed mice relative to controls (p<0.0001, Fig 4B), indicating an accumulation of MSN in the embryonic portion of the placenta.

For late-stage (GD14) injections, MRI scans were performed within 3 hours of injections. Significant hypointensity in T_2_-weighted MRI indicating accumulation of MSN was observed for both spongiotrophoblast and labyrinthine layers of MSN mice compared to controls (Fig 3C). The negligible differences at the interface between the placental layers (Fig 4A) as well as in the embryos themselves indicate the MSN remain in the placenta and do not reach the embryo.

### Ultrasonographic detection of early gestational injections

**Fig 5:**
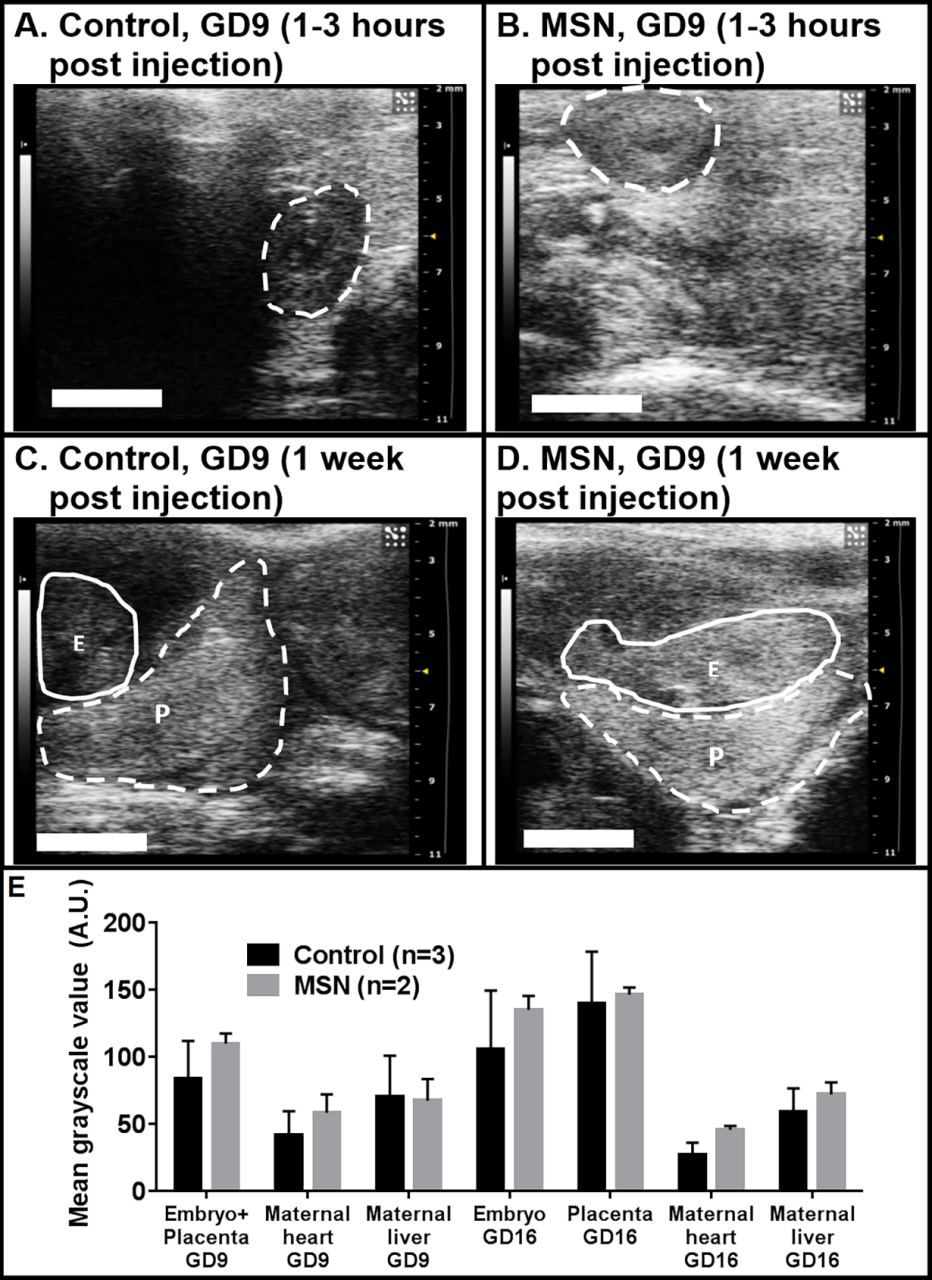
Ultrasound results of gravid mice exposed to MSN injected early-mid gestation (GD 9). Within 3 hours of tail vein injection of normal saline (A) or MSN (B), placentas/embryos (dashed lines) are noticeably more intense (whiter) for MSN-exposed mice relative to control mice, indicating an accumulation of MSN. Seven days after injection (C,D), the embryos (solid lines, E) of MSN mice are more intense, while the placentas (dashed lines, P) have a similar intensity. In the plot (E), the mean normalized grayscale values for each volume are shown. Scale bars indicate 3 mm. Error bars indicate standard deviations; pairwise comparisons between control and MSN mice were not statistically significant (p>0.05).

Because ultrasonography is a significant clinical tool for maternal-fetal diagnosis, we functionalized our MSN to produce a visible signal with the addition of trifluoropropyl moieties. These moieties, as well as the increased density of MSN relative to soft tissue, generate high intensity speckle when viewed with ultrasound [40,41]. Initial measurements of ultrasound scans were performed on GD9, with 3 control mice and 2 MSN-exposed mice, with scans being performed 3 hours after injections. At this early stage, the fetus and placenta are about 5 mm in diameter and not differentiable into two separate volumes. Therefore, the entire mass was treated as one volume. When the data from the 3 control mice and 2 MSN mice were pooled, the intensity of the combined embryos/placentas was noticeably brighter after MSN exposure compared to the controls (Fig 5A and 5B). With 2 MSN mice and 3 control mice, the difference was not statistically significant. The maternal livers in MSN and control mice had equivalent grayscale intensity, indicating consistent normalization of imaging parameters (Fig 5E).

Follow up scans performed on the same mice one week later (GD16) showed equivalent ultrasound intensities in the placentas of MSN and control mice (Fig 4C and 4D), with considerably brighter intensities in the MSN-exposed embryos (134.9±10.3 A.U) than in the controls (105.6±43.6). With 2 MSN mice and 3 control mice, the difference was not statistically significant. Thus in early gestational stage injections, particles translocate relatively freely through the placenta and reach the fetus.

### Ultrasonographic detection of late gestational injections

**Fig 6:**
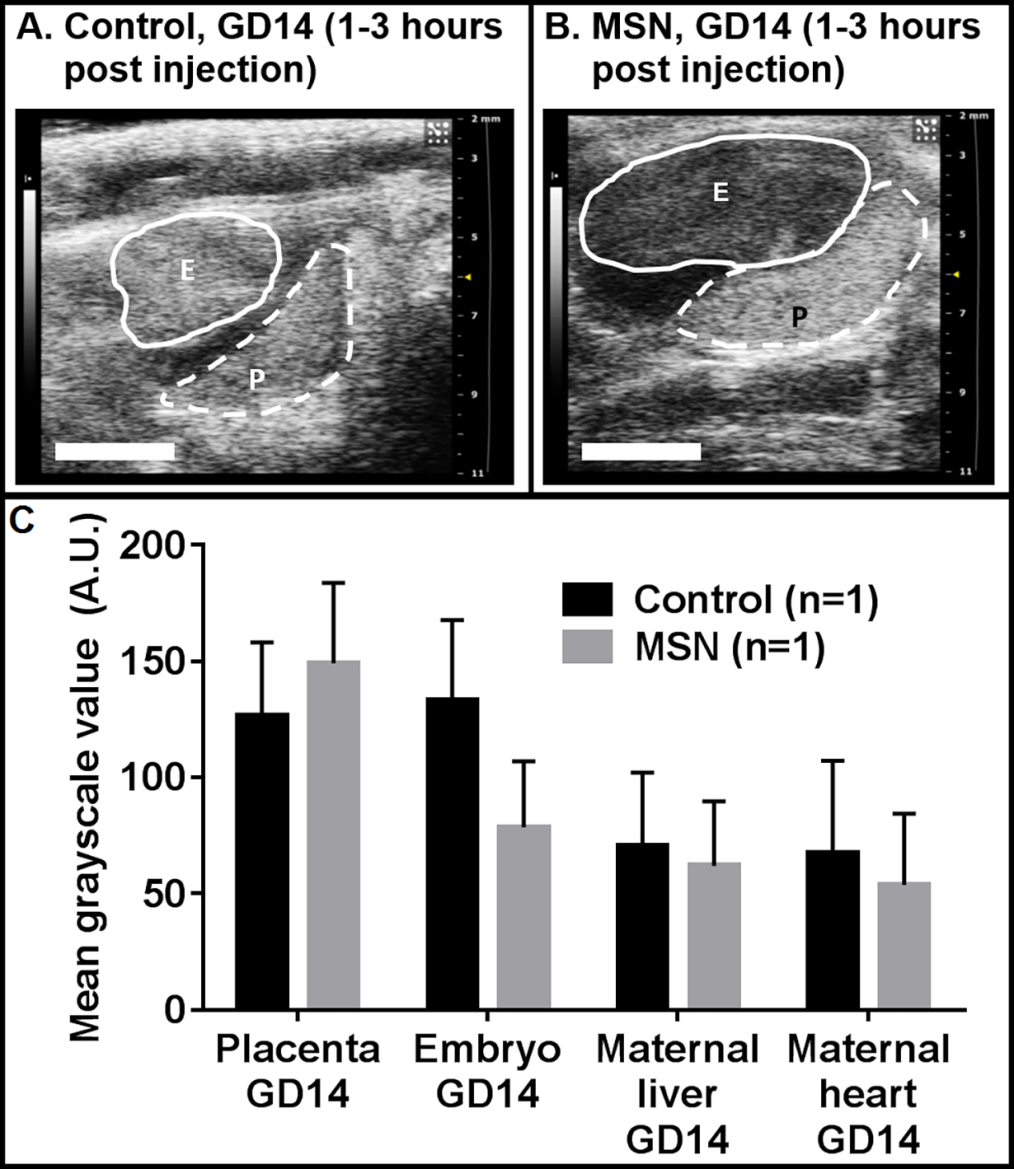
Ultrasound results of gravid mice exposed to MSN injected late in gestation (GD 14). Within 3 hours of tail vein injection of normal saline (A) or MSN (B), placentas (dashed lines) are noticeably more intense (whiter) for MSN-exposed mice relative to control mice, indicating an accumulation of MSN, while embryos (solid lines) are less intense in MSN-exposed mice. In the plot (C), the mean normalized grayscale values for each volume are shown. Error bars indicate standard deviations of grayscale intensities within each volume of interest.

Following MRI scans, gravid mice injected with MSN or normal saline on GD14 were scanned using 30 MHz ultrasound (Fig 6). The maternal circulation (heart and liver) was equivalent for MSN and control mice. The intensity of the placenta of the MSN-exposed mouse was higher than the control, while the embryo was lower, indicating a placental, as opposed to embryonic, accumulation of particles. The images show equivalent cross-sections of the placenta, while the embryos are at differing orientations. In the control image, the embryo was scanned closer to its edge, whereas the MSN-exposed embryo was scanned near its mid-sagittal plane. The mechanical properties (elasticity) of the tissue at their respective locations likely account for the difference in intensity between the two embryos.

## Discussion

In this study, we evaluated a novel, multifunctional MSN for real-time and non-invasive MRI and ultrasound detection of maternal-fetal circulation. This approach provides a unique solution for the documentation of time-dependent changes in placental transport of MSN. Prior to making these evaluations, it is important to accurately characterize the particle size. Our TEM measurements indicate a diameter of less than 100 nm for MSN alone (Fig 1B) and 100–200 nm for Gd_2_O_3_-MSN (Fig 1C), while DLS provides a hydrodynamic particle diameter of 187 nm for Gd_2_O_3_-MSN and 227.8 nm for the complete TFP-FITC-Gd_2_O_3_-MSN. When considering DLS results, it is important to remember the impact of instrument sensitivity to high or low particle concentrations as well as the interaction between the particle and dispersant, forming the Stern layer. The hydrodynamic diameter will always be larger than the “dry” particle diameter as measured by electron microscopy, but the magnitude of the difference varies greatly between samples and dispersants. Lin et al report TEM diameters of 42 nm and 25 nm for their modified MSN particles, with hydrodynamic diameters of 60 and 40 nm, respectively, an increase of 43% and 60% [42]. Qiao et al recently report TEM diameters of 55 and 67 nm, respectively, for non-coated and gel-coated MSN. The non-coated MSN had a hydrodynamic diameter of with respective hydrodynamic diameters of 70 nm, an increase of 27%, while the gel-coated MSN had a hydrodynamic diameter of 180 nm, an increase of 157% [43]. Rafi et al report a TEM diameter of 40 nm for their MCM-41 MSN, and DLS indicate a particle diameter of 600 nm, a 14-fold increase in measured size [44]. These significant increases in measured size are attributed to interactions between the aqueous dispersant and hydrophilic gel coatings. The aggregation of particles in the bloodstream is likely to affect particle diameter as well, though this would be difficult to model *in vitro*. With respect to our particles, we have previously shown circulation without significant or deleterious aggregation [30]. In short, while DLS provides a high sample throughput and low cost of operation, electron microscopy is the gold standard for true precision measurements of particle size.

To test particle stability, DLS measurements have been taken of dry powders and suspensions immediately after synthesis and following up to two years of dry storage. For long-term storage, dry powders are preferable to aqueous suspensions to increase the stability of the functional groups attached to the particles—i.e., peptides, fluorophores, and the trifluoropropyl moiety. Particles are resuspended in ethanol and sonicated using an ultrasonic water bath operating at 42 kHz and 35 W for 5 m immediately prior to DLS. Under these conditions, we have seen no evidence of particle aggregation after long-term storage.

While study of human placenta is largely limited to full-term ex vivo models, the United States FDA requires drugs to be tested on one rodent and one non-rodent placental model (4). To meet these stringent requirements, mouse and rabbit placentas have been deemed an analogous model for early-term placentas. In both of these models, the placenta undergoes histologic changes which makes it more restrictive to the passive transfer of molecules. This occurs on about gestation day (GD) 11 in mice and GD13 in rabbits, and corresponds to Carnegie stage 13, approximately 30 days in terms of human gestation [45].

We reason that this limitation in nanoparticle uptake may stem from the labyrinth layer of the placenta. In mice, the maternal circulation is in direct contact with a single layer of mononuclear trophoblasts surrounded by a dual layer of syncytiotrophoblast cells that line the fetal capillaries [46]. It is in this cell layer that PEGylated gold nanoparticles have been blocked in human placenta [47]. Given the role of these cells in strictly regulating nutrient uptake into the fetal capillaries, we expect there to be an accumulation of nanoparticles in or around the dual layer of syncytiotrophoblast cells. Confirmation of this hypothesis may be possible through histology or by quantifying the MRI intensity levels of a constant volume section that passes though the boundary between the spongiotrophoblast and labyrinth layer. Presence of an enhanced signal from a MR paramagnetic contrast agent would allow us to quantitatively identify the fetal and placental uptake before and after this gestational event.

It is increasingly evident that certain forms of nanomaterials may have potential reproductive toxicity [8,9,48]. Despite these rising concerns, relatively few scientific publications link nanoparticle exposure and teratology [4,21]. Lack of an accepted model is to be blamed for the paucity of studies of potentially detrimental effects to proper fetal development. Major anatomical and histological difference of the placental barrier in human and laboratory animal make it difficult to compare the transport of nanomaterials from mother to the developing fetus. However, emerging technologies including nano-scale contrast agents and novel imaging techniques in live animal models may pave the way for fundamental discoveries which may lead to clinical applications. The purpose of this paper is to establish a reasonable link between *in vivo* models and largely untenable human testing.

Our findings confirm that there is a differential flow pattern of particles and accumulation *in utero* according to the time of injection: early gestational stage or late gestational stage. For early-stage (GD9) injections, MRI signal is hypointense in both placenta and embryo of MSN mice compared to their sham injected counterparts (Fig 2A and 2B). The extent of the hypointensity varies from mouse to mouse. We attributed this variation to the success and completeness of the injections, that is, how much material was infused into the blood circulation versus the tail interstitium. While it is true that the results are not statistically significant, the arrows highlight hypointensities which are considerably greater in MSN-exposed embryos (and not placentas) than in the controls. Due to the small volumes involved, we chose to carry out a follow-up on the same mice one week later (GD16; Fig 2C and 2D). In these follow up scans, the observed hypointensity (arrows) is specific to the fetal spongiotrophoblast, and not the mixed labyrinthine layer, of the placenta. This data leads us to conclude that the MSN cleared the placenta following the early injection, entered the embryonic circulation, and eventually accumulated in the spongiotrophoblast. The ultrasound data (Fig 5) supports this conclusion via the increased intensity of the GD9 embryo, both immediately after the injection (Fig 5B) and 1 week later when the placenta and embryo are well-resolved (Fig 5D). So in mice, during early gestation the transplacental diffusion of small particles is lenient. It has been reported that even larger particles, such as 300 nm polystyrene, will pass through [7]. In contrast, the GD14 (late gestation) placentas are hypointense relative to the GD9 placentas (p<0.0001) and even compared to the GD14 embryos themselves (p<0.0001). At this stage, a similar degree of hypointensity is noticeable for both layers following particle injection. This may point out to the retention of particles in the maternal circulation and the fetal endothelium, but not entering the fetal circulation.

To further confirm these results we performed ultrasound experiments. Our ultrasonographic measurements generated in parallel strongly corroborate MRI data. Qualitatively, both experimentations are in agreement concerning signal intensity in the presence of MSN. Quantitatively, however, statistical significance was not achieved. Methods to improve quantitative statistical analysis of contrast-enhanced ultrasound images is an active area of research, with no singular established guideline. The ultrasound output image is sensitive to variables including blood flow, tissue elasticity, and the pressure applied by the operator on the transducer. These factors combined with the variance inherent in scattered waves make quantitative and statistically significant data difficult to acquire [49]. From a clinical standpoint, a qualitative ultrasound is often sufficient (i.e., visualization of a fetus, guidance of a catheter, diagnosis of tumors, faulty heart valves, etc.). In the case of this study, our ultrasound images are corroborated by sharper MRI studies at equivalent time points. Data for both methods are congruent with regard to the clear indication of preferential localization during various gestational ages. Taken together, results provide strong evidence in support of our hypothesis: between GD9 (early gestation) and GD14 (late gestation), the placenta in our mouse model, becomes more exclusive as gestation progresses, to the point of excluding MSN from the embryo. So in mice, during late gestational period the transplacental diffusion of small particles is segregated and more selective.

We previously used a murine maternal-fetal exposure model to evaluate the toxicity of MSN in both the adult and developing organism [30]. In developing mice, toxicity is often reported by underweight embryos/placentas or a smaller than normal litter size. To identify whether MSN exposure had an adverse effect on fetal/placental growth, at GD9, GD14, and GD16, the MRI-measured volumes of embryos and placentas were previously compared in MSN-exposed mice versus controls and found not to be significantly different. In a follow-up excision of uteri on GD17, embryos and placentas had equivalent weights between treatment groups as well [30]. Further, we did not observe elevated levels of inflammatory cytokines or reactive oxygen species in either the mothers or embryos, and histopathology of the maternal livers and kidneys also showed no remarkable changes resulting from MSN exposure.

A careful review of the literature found that studies of MRI-based measurement of gestation in the mouse are limited to *ex vivo* examples for the purpose of creating anatomical atlases of the developing mouse, and thus not useful for comparison. Instead, we identified a study which measured the growth of embryos and placentas in the new world mouse (Necromys lasiurus), using 3-dimensional stereology [50]. Our MRI indicated weights of MSN-exposed and control embryos follow the measurements reported therein, while placental measurements vary considerably. This is likely due to significant differences between N. lasiurus and M. musculus with respect to placentation. Principally, the average litter size of N. lasiurus is only 3–6 pups, whereas M. musculus typically has 6–8 pups per litter. The authors also note that by the end of gestation, placentas of M. musculus have a 3-fold larger volume than those of N. lasiurus. In addition, placenta volumes for mice at early gestation were not provided, while the earliest data for N. lasiurus are from GD10.

On a technical note, unlike CT, PET, and ultrasound, the output of an MRI scan is unitless, with relative grayscale values related to the frequency response of the tissue to a particular pulse sequence. Thus, it is important to implement a consistent normalization strategy for comparison of tissues between different subjects and scans. To date, there is no agreed-upon methodology for normalizing MRI scans [51]. Given the inherent variability between scans, normalization steps are important. In this study, we calculated the mean gray scale value based on fat (set as 100 or 1000) and interpolating all values in the image between 0 and the arbitrary value. *In vivo*, fat is minimally vascularized, thus unlikely to be acutely affected by the intravenous injection of contrast agents [52].

Implementation of this normalization strategy allowed us to quantitatively compare grayscale values between MRI scans acquired with the same parameters, and confirm the findings of time-dependent changes in transport properties. To a lesser degree, the grayscale values followed similar trends in sham-treated mice. This is likely due to mild edema altering the MRI signal [53] caused by increased blood volume following the infusion of 0.1 mL saline.

Because of the time required to scan each mouse in MRI, some mice were scanned relatively soon after injections, while others were scanned 3–4 hours following injections. Scanning of each mouse and injection has to be orchestrated to minimize lag time, which may obscure the results. To rule out this possibility, MSN-exposed mice from GD9 (n = 23 embryos from 3 mothers) and GD14 (n = 29 embryos from 4 mothers) were pooled, and the normalized MRI intensity was plotted against the time between injections and MRI scans. The supporting statistical analysis of these results confirm our hypothesis; slopes near zero and low R-squared values, indicating no significant correlation between MRI signal and the time between the injection and the scan. Conversely, the strongest correlation obtained were observed with placentas on GD9 with an R-squared value of 0.3286. With a slightly negative slope, these results suggest a slight decrease over time in the normalized MRI intensity and an indication of a time dependent accumulation of particles in the placenta. Furthermore, the particulate trans-localization between the layers appears to quantitatively correlate with gestational maturity of the fetus.

As further knowledge of the placenta is gained, a clearer image of its role is coming into focus. With the help of novel technologies, a more accurate determination of its pivotal function will be ascertained. The placenta is a lot more than just a mere passive filter but rather a complex and essential organ for the proper development of the fetus. For instance, in addition to active and passive forms of transport, the placenta can be thought of as a glandular organ, secreting a number of growth factors and other hormones [54]. Yet, the embryo is often exposed to a number of toxicants ingested by the mother consciously or via unwitting environmental exposure. Alcohol, narcotics, and the byproducts of cigarette smoke or pollutants and chemical pesticides have been shown to cross the placental barrier and undoubtedly affect fetal development [12,15,55,56]. The pivotal role of the placenta as a filter or de-toxicant remains largely undocumented. In this study, we introduce a potentially new tool in the form of a non-toxic MSN to help the real-time and non-invasive evaluation of exposed embryos. This is evidenced by cumulative data that showed equivalent birth weight, placenta weight, and low levels of inflammatory cytokines relative to control embryos.

## Conclusions

As industrial and medical use of nanomaterials increases, so does the likelihood of embryonic exposure to these materials via the maternal circulation. As a result, the role of the placenta in translocation of nanomaterials from mother to developing fetus is an area of high research interest. To our knowledge, this is the first study to demonstrate the benefits of a tool for minimally-invasive and real-time evaluation of maternal-embryonic circulation using an *in vivo* small animal model. In this study we showed that 2 built-in tagging compounds within a small molecule allow for both MRI and ultrasound investigations (a testament to the multifunctionality of the particles which have been verified and actively used in *in vivo* experimentation with all modalities described). Additionally, the evidence for the use of MSN containing fluorescein was previously presented [30]. In brief we previously showed using these particles that individual cells could be labeled fluorescently and visualized over 4–5 generations. We confirm some of the earlier findings that show time-dependent changes in the transfer of materials across the placental barrier. Indeed, prior to GD11 in the mouse, equivalent to day 30 of human gestation, we observed signal from MSN in the embryonic circulation, whereas after this key points in gestation, signal intensity changes are only observed in the placenta. These findings usher in two novel study paradigms. In the first, the MSN are used *in vivo* to evaluate the trafficking of conjugated toxicants. In many environmental toxicological studies, a mechanistic link between an environmental insult and a biological response is elusive. By conjugating the MSN with toxicants, we can visualize their binding and correlate its effect on its microenvironment over time. In the second paradigm, MSN act as scavengers of circulating toxicants. In the event of exposure (such as a pregnant woman exposed to drugs of abuse or environmental insults), a bolus of MSN bearing the reagents needed to neutralize the insult is delivered, while the MSN can be visualized in real-time. We are currently involved in implementing the proof-of-concept of these studies. As an added advantage, the porous capacity of the MSN for loading and potentially delivery of biologically active cargo makes it a candidate for diagnostic/therapeutic applications.

## Supporting information

S1 Fig Characterization of mesoporous silica nanomaterials (MSN). Powder x-ray diffraction (XRD) of Gd_2_O_3_-MSN confirmed ordered pores in the diffraction pattern of the as evident by an intense d_100_ peak at 2.5 2θ, and well resolved d_110_ and d_200_ peaks at 3.83 and 4.40 2θ, respectively. This diffraction pattern is consistent with P6 mm hexagonal symmetry characteristic for MSN.
(TIF)

S2 Fig Ultrasound raw images. Mice received injections of MSN (n = 2) or vehicle (control; n = 3) on GD9 and were subsequently scanned on GD9 and GD16. Examples of embryos/placentas from each mouse are presented.
(TIF)

S1 File MRI raw images. S1_File.zip contains all MRI data used in this manuscript, organized by gestation day (GD9, GD14, or GD16) and by MSN versus control. Scans were T2-weighted axial MRI and files are nifti (.nii) format.
(ZIP)
