# Supplementary figures and images for: Multifunctional nanoparticles for real-time evaluation of toxicity during fetal development
Source: PLoS One. 2018 Feb 8;13(2):e0192474. doi: 10.1371/journal.pone.0192474 (PMC5805299; doi:10.1371/journal.pone.0192474)

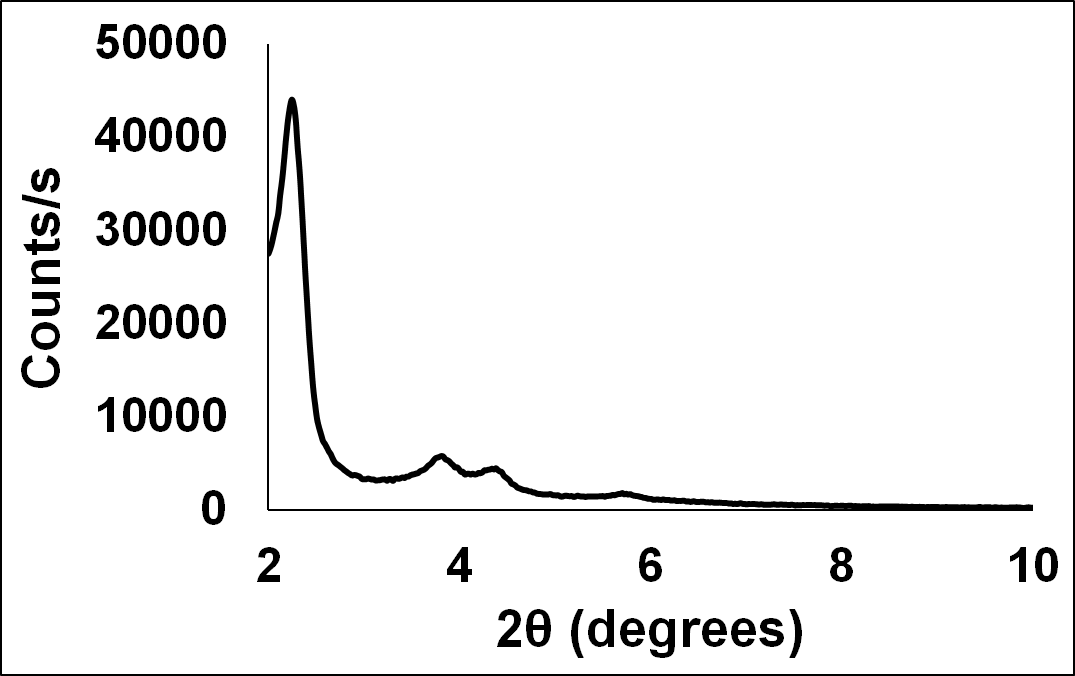

Supplement: S1 Fig — Powder x-ray diffraction (XRD) of Gd2O3-MSN confirmed ordered pores in the diffraction pattern of the as evident by an intense d100 peak at 2.5 2θ, and well resolved d110 and d200 peaks at 3.83 and 4.40 2θ, respectively. This diffraction pattern is consistent with P6 mm hexagonal symmetry characteristic for MSN. (TIF) [file pone.0192474.s001.tif]

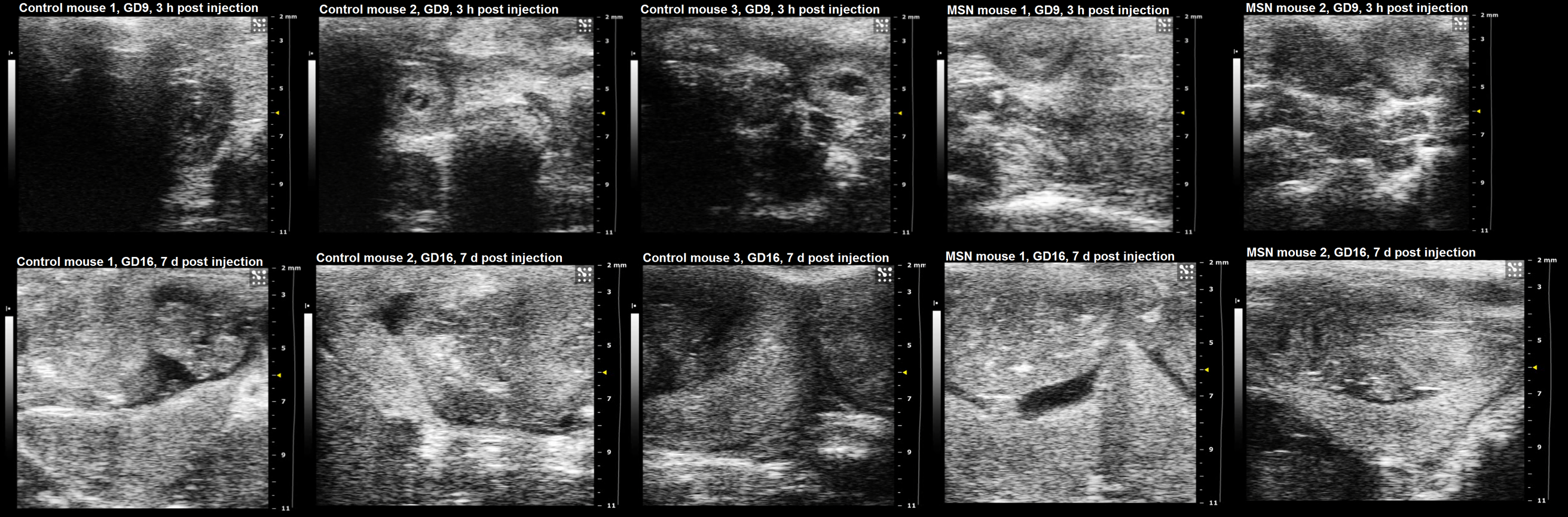

Supplement: S2 Fig — Mice received injections of MSN (n = 2) or vehicle (control; n = 3) on GD9 and were subsequently scanned on GD9 and GD16. Examples of embryos/placentas from each mouse are presented. (TIF) [file pone.0192474.s002.tif]
